# Service reconfiguration in the department of hand surgery during the UK COVID-19 lockdown: Birmingham experience

**DOI:** 10.1136/postgradmedj-2020-139280

**Published:** 2021-01-27

**Authors:** Natasha Emma Picardo, Harriet Walker, Qureish Vanat, Bafiq Nizar, Tomas Madura, Rajive Jose

**Affiliations:** Hand Surgery, UHB NHS FT, Birmingham, UK; Hand Surgery, UHB NHS FT, Birmingham, UK; Hand Surgery, UHB NHS FT, Birmingham, UK; Hand Surgery, UHB NHS FT, Birmingham, UK; Hand Surgery, UHB NHS FT, Birmingham, UK; Hand Surgery, UHB NHS FT, Birmingham, UK

**Keywords:** health & safety, health policy, human resource management, risk management, plastic & reconstructive surgery, orthopaedic & trauma surgery

## Abstract

In early 2020, the COVID-19 pandemic swept through the UK and had a major impact on healthcare services. The Birmingham hand centre, one of the largest hand trauma units in the country, underwent a dramatic service reconfiguration to enable robust and safe provision of care that would withstand the peak of the pandemic. Streamlining our service significantly reduced patient footfall and hospital admission while preventing intra-hospital viral transmission. Many of the changes implemented have been kept as permanent adjustments to our practice as this new model of care yields higher patient satisfaction and efficacy to withstand the pressures of further peaks in the pandemic.

## Introduction

In December 2019, a cluster of cases of viral pneumonia was reported in Wuhan, Hubei Province in China.^1^ In early January 2020, a novel coronavirus (SARS-CoV-2) was confirmed to be the causative pathogen,^2^ which was transmitted via respiratory droplets or through indirect contact with these secretions.^3 4^ Most patients had a mild illness or were asymptomatic; however, approximately 13.8% required secondary care support for severe respiratory symptoms and 6.1% were critical needing ventilation.^5^

The rapid spread of this disease around the world prompted the WHO to declare a pandemic on 11 March 2020.^6^ To reduce the rate of transmission, the UK government imposed a lockdown on 23 March 2020, which lasted for 10 weeks until 31 May 2020. During this lockdown period, the number of reported deaths in the UK attributed to COVID-19 increased from 331 to 37 435.^7^

The Queen Elizabeth Hospital (QEH) is part of the University Hospitals Birmingham Foundation Trust. It is based in the West Midlands and houses the largest critical care unit in the country with a permanent capacity of 125 beds. Of the local urban population, 40.2% are from Black Asian and Minority Ethnic (BAME) groups who have been particularly affected in the pandemic. Since the outbreak of COVID-19, our trust has treated the highest number of confirmed patients with COVID-19 and had the highest number of COVID-19-related deaths in the UK.^8^

The QEH is also home to the Birmingham Hand Centre (BHC), one of the largest adult hand trauma units in the country with a referral population of 5.5 million, representing 8% of the UK population. The department comprises 13 consultants, 7 peri-CCT senior fellows, 9 middle grades and a shared pool of core trainees and specialty trainees from an orthopaedic and plastic background (see online supplemental glossary). Hand surgery is regarded as its own specialty with separate on call, clinical service lead and governance structure.

At the start of the lockdown period, our department underwent complete reconfiguration to streamline patient care. At the forefront of the agenda, the aim was to have a greater front door consultant presence to expedite decision-making and definitive treatment while reducing footfall and maintaining the safety of staff and patients. Our department’s response to the evolving crisis is detailed below, which we feel is robust enough to withstand further peaks in the pandemic. The efficacy of our new working practice has prompted us to permanently alter our working practice for the post-COVID-19 era.

### Acute hand referrals pre-pandemic

Prior to the COVID-19 pandemic, an acute hand trauma clinic (hot hands clinic) existed which was staffed by on-call junior doctors and a trauma nurse from 08:00 to 17:00. The clinic was based in an ambulatory care unit and received local referrals from our own emergency department as well as regional referrals via a locally developed electronic Network On call Referral system (NORSe).^9^ This software system enables electronic messaging between our hospital and referring hospitals as well as the confidential and secure exchange of patient data, photographs and radiographs. Patients accepted through this pathway would be seen and assessed and small cases such as minor wounds, extensor tendon repairs and nailbed injuries operated on in a neighbouring minor operations theatre, which was staffed by operating department practitioners and nursing staff. The cases were performed by the on-call junior doctors under local anaesthetic, often using wide awake local anaesthesia no tourniquet (WALANT) technique.^10^ Cases needing formal surgical treatment, for example, under regional block or occasionally general anaesthesia, received initial wound washout and were discussed in the morning meeting the following day with the on-duty consultant and planned for consultant-led rolling trauma theatre lists. More urgent complex cases, for example, infections, polytrauma or those needing revascularisation or replant were seen in the emergency department by the on-call team, discussed with the on-duty consultant and admitted for emergency theatre lists. In all scenarios, patients would be assessed using a locally developed digital hand trauma assessment and management tool (eHands). This system is designed to capture accurate patient demographic data, injury mechanism, site and severity data and allows injuries to be classified and procedures to be coded. This is fundamental to tracking our departmental activity. Patient operation notes are completed using this system and sequential operations and clinic attendances can be added to follow the patient pathway through the hand service. Lists can also be generated to identify outstanding investigations and procedures. The hand coordinator, a senior nurse, was integral to service provision, planning operating lists and liaising with patients.

Following procedures, patients would receive a dressings clinic follow-up for suture removal or would make a general practitioner (GP) or practice nurse appointment. Most patients required subsequent hand therapy appointments and further medical review in a consultant-led hand clinic.

**Figure 1:**
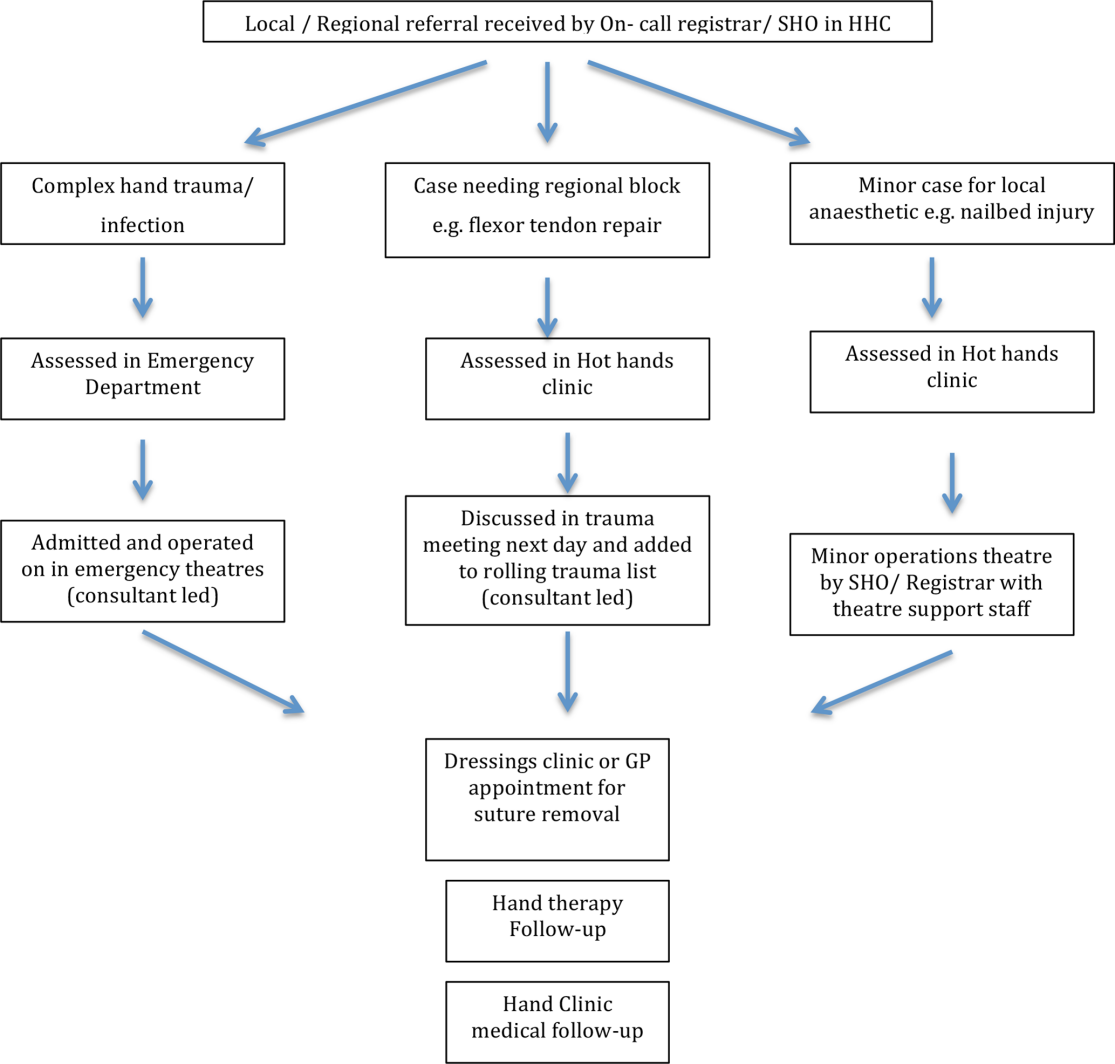
Pre-COVID-19 model of acute hand trauma management. GP, general practitioner.

This pre-COVID-19 pathway for acute hand trauma is illustrated in figure 1. Patients were mostly assessed by the hand fellow or registrar on call at the first presentation to the hand trauma service and often the management plan would change following the handover meeting the next day. Patients would then need to be contacted and in some cases brought back for a second assessment, swabs, consent, blood tests and so on. In each of the procedural pathways, patients would come into contact with a large number of healthcare workers and this further involved unnecessary patient traffic around the hospital and between primary and secondary care.

### Acute hand referrals during lockdown

**Figure 2:**
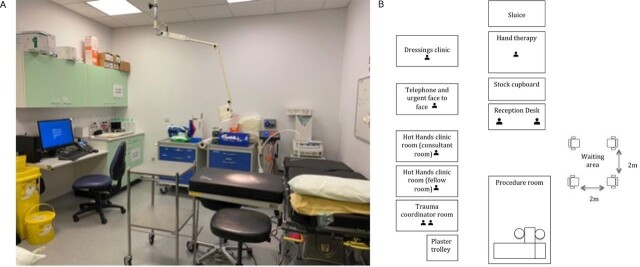
(A) Photograph of procedure room. (B) Floor plan of hot hands clinic illustrating layout and members of staff involved.

At the start of lockdown, the hot hands clinic hours were extended to 08:00–20:00 7 days a week and this was always manned by both a duty consultant and a peri-CCT fellow or senior registrar with support from a dedicated team of hand coordinators. The clinic was relocated to a COVID-19 ‘cold’ area of the hospital and a procedure room was recommissioned as a minor operating theatre. A photograph of the procedure room and floorplan of the clinic is shown in figure 2A,B. NORSe was managed directly by the duty consultant and COVID-19 risk stratification was performed by a symptom and contact-screening questionnaire. The information from NORSe referrals, including photos and radiographs allows a detailed analysis and therefore a tentative management plan to be formulated before any physical contact with the patient has occurred. This was particularly beneficial as advance planning maintained efficiency and safety, for example, by staggering patient attendances and mobilising extra staff if needed. At the point of referral, we asked that radiographs were performed and antibiotics and tetanus administered in advance of presentation to our clinic. These measures were instigated to reduce further footfall around our hospital.

Patients without COVID-19 symptoms or contacts (assumed COVID-19 negative) were triaged to assessment in the hot hands clinic (cold pathway). Patients attended clinic alone and wore face masks as did all clinic personnel. There was a contact-free check in kiosk available in clinic for expected patients to report to eliminating exposure with reception staff. All patients were assessed by a consultant and peri-CCT fellow using the eHands system. Where possible, a ‘see and treat’ principle was adhered to whereby procedures were undertaken immediately after assessment in the neighbouring procedure room under WALANT. As time went on, we became more confident in our use of WALANT technique, and at the peak of the pandemic, we undertook nerve and simple tendon repairs as well as digit terminalisations in the procedure room. In some cases, we had to modify our standard operating practice to minimise patient and staff risk. Examples included the manual passing of a hypodermic needle across a distal phalanx fracture for bony stabilisation to avoid the aerosol generation of K- wiring. All patient noting and management decisions were copied into the patient’s electronic notes with a special note to mention that the patient had been assessed and treated during the coronavirus pandemic.

The performance of small cases under local anaesthetic in a procedure room had a multitude of benefits. It reduced the need for regional blocks and general anaesthesia at a time where many anaesthetists and theatre support staff were redeployed to ITU. Cases that we would have normally admitted such as dog bites could be washed out early and sent home on antibiotics thus reducing the need for inpatient beds and preventing hospital-acquired infections.

A supply of antibiotics, analgesics and tetanus injections was maintained in the hot hands clinic, which reduced patient traffic to the outpatient pharmacy. Furthermore, absorbable sutures were used in wounds to reduce clinic appointments for suture removal, particularly at the peak when access to primary care was limited. We were able to apply plaster casts in the procedure room to prevent the footfall through the hospital fracture clinic where this was traditionally undertaken. Follow-up imaging was only performed when there was likely to be a significant change in management.

In certain cases, we gave the patient dressings to change at home and even cast removal instructions. In all these scenarios good communication with the patient was key to prevent the development of complications at home and the clinic contact number was provided.

A constant hand therapist presence in clinic was also vital in avoiding unnecessary hospital attendances. Patients could see the hand therapist at their first assessment visit and were instructed on how to rehabilitate from day one. In addition, if needed, a splint for later use could be fabricated at the same time, further illustrating the ‘one stop shop’ model of care. For therapy follow-up, a home delivery service for splints and dressings was also employed.

Assumed COVID-19 negative patients needing admission and an emergency operation were assessed in hot hands clinic or the emergency department ‘cold’ area. They were then admitted to a COVID-19 ‘cold’ ward in the hospital and operated on in emergency theatres at the earliest opportunity.

Cases that were not emergencies and were not suitable for local anaesthetic (eg, multiple flexor tendon repairs) were assessed in hot hands clinic then sent home with an appointment for a rolling hand trauma list at our sister hospital, the Royal Orthopaedic Hospital (ROH) under regional block within forty-48 hours. The pathway was part of the service reconfiguration and replaced elective surgery capacity. These lists were staffed by consultant hand surgeons who, due to risk profiling were advised against working in the hot centre at QEH. As elective operating had all been suspended in keeping with national guidelines,^11^ we were able to use this hospital as a ‘cold’ operating site. Again in some cases we had to modify our usual operating practice to keep this site safe—for example, avoiding aerosol-generating procedures such as Kirschner (K)-wiring and using handheld dermatomes instead of electric ones for harvesting skin grafts.

Patients with confirmed COVID-19 and those with symptoms or positive contacts (suspected COVID-19 positive) were assessed in the emergency department ‘hot’ area by the duty consultant and fellow. If admission and/or a procedure were necessary, they were admitted to a COVID-19 suspected or confirmed ward and their case undertaken in emergency theatres. Precautionary theatre measures included the provision of full personal protective equipment (PPE) with supervised donning and doffing, one-way human traffic, avoidance of general anaesthesia and deep cleaning between cases. At the start of the lockdown period, before access to rapid COVID-19 swab results was available, all procedures involving aerosol generation, for example, K-wiring were also undertaken with full PPE regardless of patient symptoms or contacts. Patients with suspectedCOVID-19 were subsequently stepped down from the ‘hot’ pathway if they had no COVID-19 symptoms, negative radiological imaging (chest radiograph±CT) or if available, a negative SARS-CoV-2 test. In all cases, patients were treated in accordance with the WHO and UK specialist society guidelines.^12–14^

**Figure 3:**
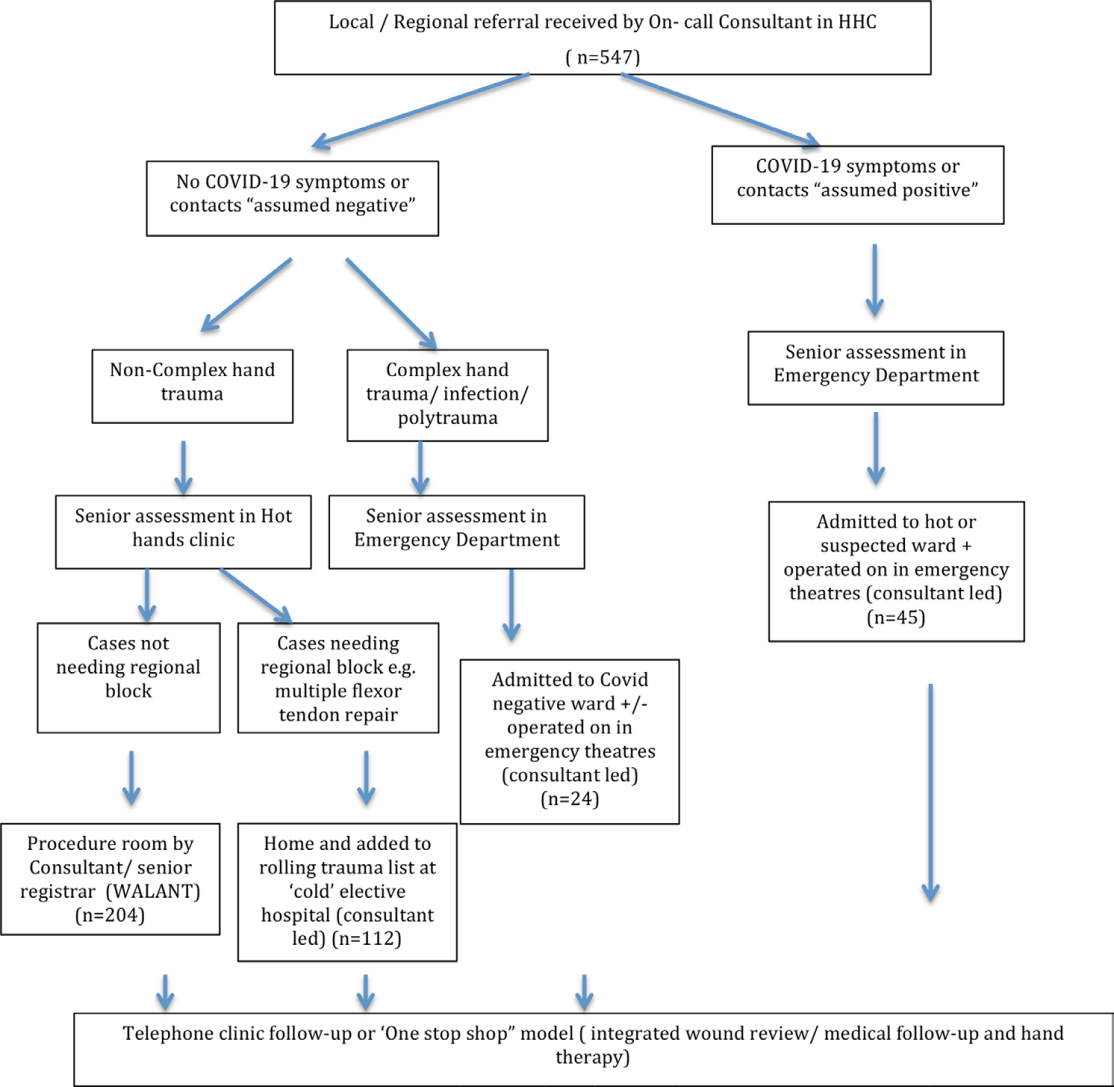
COVID-19 pathway for acute hand trauma referrals. WALANT, wide awake local anaesthesia no tourniquet.

A summary of the COVID-19-modified acute hand trauma pathway with the patient numbers is shown in figure 3. In all cases, follow-up was streamlined so that patients would be able to have their medical follow-up, wound check and hand therapy in the same clinic on the same visit.

## Changes to staff working patterns

To meet the needs of our 7-hour service, consultants and senior registrars were reallocated to a new working pattern. The department was split in half (team A and team B) and a 4-hour on, 4-hour off rota pattern was employed with 12–13 hour shifts to allow a structured handover at 08:00 and 20:00. There was a second duty consultant and registrar available as backup. This reconfiguration was essential as we lost many junior members to intensive therapy unit (ITU) redeployment at the start of lockdown and we needed a system supportive enough to be flexible around predicted high staff absence rates from sickness and self-isolation.

Generally, team A doctors did not come into contact with team B doctors. This segregation was designed to allow easy contact tracing in case a team member should become unwell and avoid cross contamination with the other team who could then step in for duty.

The on-duty consultant was resident in the hospital overnight, taking referrals and seeing ward patients. Between the hours of 20:00 and 08:00, only emergency patients were seen, others were given a hot hand clinic appointment the following day. In this way, every referral had a consultant response around the clock.

As in pre-lockdown, the trauma coordinator role was essential in planning operating lists and liaising with patients at home. During this time, the role was modified however to include new patient assessment in clinic, preoperative COVID-19 screening, preparing the procedure room and assisting in minor operations. Every member of the team changed into scrubs and a face mask at the start of each shift and back into normal clothes at the end. Social distancing guidelines were adhered to during all clinical activity.

The traditional in-person morning meeting was abolished in favour of a daily video conference involving all consultants to discuss patients and plan each day’s activity and any identified operational issues. Complex patients were also discussed to gain departmental consensus on optimal management planning. This enabled all consultants to remain familiar with the activity of the department even when not on duty or allocated to duties at a different site and facilitated more effective paper-free ward rounds where presence on wards was minimised. Ward rounds took place twice a day to address clinical issues, prevent complications and expedite early discharge.

Study leave and annual leave was cancelled at the start of the pandemic in response to national guidelines. To maintain high training standards and facilitate work rotas, consultants provided regular online teaching sessions from home or work. Junior doctors could log into these sessions as and when clinical commitments allowed and some of these took place outside of work hours. Peri-CCT hand fellows used this opportunity to work towards the Diploma in Hand Surgery. Towards the end of lockdown, annual leave was allowed once more in a phased manner alleviating staff fatigue.

### Elective activity

Prior to the pandemic, our department ran approximately 20 consultant-led clinics per week across two sites and 13 elective operating lists. During lockdown, elective surgery was suspended in line with national guidelines. All follow-up outpatient appointments were reviewed and patients were allocated into the following categories:

A/Patients suitable for telephone consultation (the vast majority).

B/Patients unsuitable for telephone consultation but low risk so could be postponed—secretaries kept a list of these patients.

C/ Patients with urgent problems or at risk from deferring their appointment—offered a formal outpatient appointment.

Each day a designated consultant on general clinic duty undertook telephone consultations for group A patients and face-to-face appointments for group C patients. New elective hand surgery referrals were not suitable for telephone consultations and hence were mostly deferred, with only the most urgent seen face-to-face. By alternating telephone appointments with face-to-face appointments, we could further reduce patient congestion in clinic and reduce the time from registration to consultation. On the occasion that we would have more than one patient in the waiting area, social distancing was observed and mask wearing was mandatory.

### Outcome of service reconfiguration

To monitor our department’s activity, we concurrently undertook a single centre observational cohort study. We used the eHands database to identify all patients referred and assessed in our unit during the 10-week lockdown period. The database was referenced against the clinical records to access laboratory results, subsequent outpatient clinic letters and morbidity data. Patients with discharge to GP or without a 30-day follow-up in our service were contacted by telephone to identify complications. Datasets for the same 10-week periods from 2016 to 2019 were also analysed as a comparison.

During lockdown, we received 547 new hand trauma referrals in our department, a 45% reduction compared with the same period in 2019.

During lockdown, the patient initial assessment took place in the hot hands clinic in 80.8% of cases, with emergency department (14.8%), ward referrals (2.7%) critical care (0.3%) and other sites including theatre (1.3%) accounting for the remainder.

The assessment was undertaken by a consultant in 70.2% and a senior hand fellow in 17.92% compared with 4% and 23% in 2019, respectively.

Of these patients, 70.4% underwent their definitive procedure at the time of initial assessment compared with a mean of 50.1% in the same time period between 2016 and 2019. In total, 424 surgical procedures were performed in 385 patients, a mean of 1.1 procedures per patient (1.09 in the historical cohorts from 2016 to 2019). In 17.9% of cases surgery was undertaken in emergency theatres, with 53% and 29.1% occurring in the procedure room and cold elective hospital site (ROH), respectively. Thirty-three patients had more than one operation, 29 had two operations, 2 had three operations and 1 each had four and five operations.

Of the patients admitted for emergency theatre lists (hot pathway), 24 were symptom negative and 45 were symptom positive, although in 40 of these, the RT-PCR test was negative. Five patients were COVID-19 positive and therefore treated on the ‘hot pathway’ exclusively. No patients treated on the cold pathways subsequently developed COVID-19 symptoms or had a positive swab test. The 30-day complication rate was 6.75% for all operated patients with an unplanned readmission rate of 1.29% (5/385) and additional reoperation rate of 3.37% (13/385). The highest rate of complications was seen in the ‘hot’ pathway reflecting the more complex injury patterns. The 30-day mortality rate was 0.36% (2/547). One of these patients was COVID-19 suspected with an open fracture that underwent washout and was awaiting definitive fixation. The other patient was COVID-19 positive and died 5 days after an amputation for infected finger bite.

### Conclusion

The reduction in new patient attendances to our hospital reflected a population who due to lockdown were less likely to sustain workplace injuries or be the victims of street crime or road traffic accidents. Patients were also less likely to present with minor injuries due to fear of contracting the virus. With the patients that did present, we needed to provide a system that treated patients in the most streamlined way possible while preventing intra-hospital transmission of COVID-19 at all costs. The importance of this was highlighted in a case from Wuhan in January 2020 where a hand surgeon and assistant contracted COVID-19 from a symptomatic patient during a 1-hour emergency hand operation. Review of this case revealed that insufficient protective measures had been employed.^15^

One of our early challenges was the unavailability of COVID-19 RT-PCR testing resulting in the reliance on symptom screening, history of contact exposure and temperature monitoring. Even when testing became more available, the estimated sensitivity of the test was reported to be approximately 70%^16^; therefore, RT-PCR testing became an adjunct to clinical screening. This, in combination with the formation of hot and cold pathways, the use of PPE and reducing hospital footfall meant that no patient contracted COVID-19 during their treatment episode.

Our hospital is fortunate to have its own software development team, meaning that we had an ideal set-up for managing patients with limited clinical contact. Through this team, we have developed NORSE and eHands and all patient records are electronic. Kiosks in outpatients allow contact-free check in of expected patients. Furthermore, our hospital has developed a secure clinical image transfer application that allows medical staff to take and send encrypted photographic images from a mobile phone which is digitally transferred to the patient electronic notes.^17^ This system allows various members of the team to assess the wound while minimising dressing changes.

A recent survey of 47 hand surgeons from 34 countries revealed a large disparity in lockdown practice in different regions of the world.^18^ Five did not change their indications for surgery at all, 3 totally stopped performing surgery and 39 modified their practice. Most of those in the latter group started to perform cases under local anaesthesia in a small theatre that they would have undertaken in the main theatre prior to the pandemic. Interestingly, in a few centres, these cases included fractures, deep infections, skin grafts and flexor tendon repairs. The types of PPE also varied considerably in both the operating room and consultation room from simple masks to eye protection and N95 masks regardless of the COVID-19 status. A limitation of the study was that it was based on a questionnaire sent out in late March, early in the UK lockdown and when the severity of the outbreak differed greatly in the countries involved. In our procedure room, it was felt that pushing the boundaries with more complex operations would have compromised patient comfort. For example, one concern is regarding the tourniquet and reperfusion pain while undertaking procedures for multidigit injuries or deep infections without a regional block. Poor lighting can also impair performance in a procedure room and full PPE can be both uncomfortable and cumbersome particularly in combination with surgical loupes. Therefore, a balance has to be struck between practicality, patient safety and staff comfort.

Of interest, giving patients more responsibility for their own wound care and rehabilitation did not appear to increase complications. The Birmingham Women and Children’s hospital produced some useful patient leaflets and advice sheets which have been published on the British Society for Surgery of the Hand website specific to the COVID-19 crisis.^14^ We have also developed written guidance, as well as rehabilitation videos, for example, following flexor tendon repair, which patients can watch in recovery. These can also be viewed at home each day while undertaking their rehabilitation further adding to the potential of remote hand therapy consultation. Educational apps such as the Chelsea and Westminster hand therapy app can also increase patient compliance and confidence.^19^

Other groups have published their own response to the crisis, offering further suggestions for streamlining care^20^ . One group, for example, used a mini C-arm set-up in their plaster room and enrolled their doctors on accelerated practical training following IRMER certification to allow independent use. This allowed manipulation of fractures and the ability to check the position in a plaster radiologically without the patient leaving the clinic.^21^ This group also implemented a National Health Service (NHS) digital technology initiative, which allowed video consultations with patients. Video consultations could provide us with an improved strategy to telephone consultations and both this and C-arm use in clinic could reduce face-to-face attendances even further.

Most of the changes we made to our practice were influenced by the British Orthopaedic Association (BOA) and British Society for Surgery of the Hand (BSSH) guidelines.^13 14^ These groups have supported treating fractures non-operatively where possible with the option of corrective osteotomy once the pandemic is over. However, it is important to bear in mind that such strategies, although pragmatic, could further add to the already considerable backlog of operations once suspended elective activity resumes. A patient with a malunion may also incur many more outpatient attendances for stiffness than one who has their fracture reduced and stabilised in the acute phase.

We have made further changes to our practice since the first wave and continue to assess our service for improvements. For example, suspected scaphoid fractures (positive clinical examination findings in the absence of a confirmed fracture on radiographs) now receive an MRI scan soon after emergency department attendance. Previously these patients would have had serial radiographs and prolonged time in plaster and regular clinical follow-up. It is thought that the early MRI diagnosis will allow these patients to be discharged or streamlined down the scaphoid fracture pathway, reducing footfall. Such a strategy has been shown by other groups to be cost effective as well as enhancing patient satisfaction.^22^

In summary, streamlining our services significantly shortened the patient journey, reduced inpatient admission and resulted in high levels of patient satisfaction. Many of the changes we are endeavouring to keep as long lasting adjustments in our practice as we hopefully emerge into a post-COVID era with the introduction of vaccinations. These measures are outlined in table 1. This will allow us to maintain a robust service should a different coronavirus strain or future pandemic occur. It could also herald a paradigm shift in patient care which if widely adopted could drastically ease the annual winter pressures on the NHS and improve efficacy and patient satisfaction.

**Table 1:** Practices we anticipate keeping post-COVID-19 and a blueprint for how our system would adapt to further pandemics

Anticipated practice post-COVID-19	Blueprint for further pandemics
Consultant/fellow front of house for all acute referrals	Switch back to team-based rota with resilience for absences
One-stop-shop model of care	Prioritise only urgent and time critical elective work
Procedure room for minor cases under WALANT	Complete segregation of elective and trauma services
Seven day service	Remote access trauma meetings
Consultant-led ward round and efficient patient discharge	Remote appointments where possible
Video/telephone consultations alternating with face to face	Use procedure room where possible
Senior clinician-led service planning	Daily strategy meetings and appraisal of resources
Absorbable skin sutures, more pt involvement in postop care	More senior nurse input as junior doctors redeployed
Senior trauma nurse training to assist in minor cases	Minimal use of general anaesthetic
Early diagnosis pathways—for example, MRI for suspected scaphoid	See patients directly after triage to offload emergency department
Permanent utilisation of face masks particularly in vulnerable patients	
Offload emergency department where possible—especially in winter	

WALANT, wide awake local anaesthesia no tourniquet.

## Supplementary Material

postgradmedj-97-532-DC1-inline-supplementary-material-1
