# Supplementary material for: Service reconfiguration in the department of hand surgery during the UK COVID-19 lockdown: Birmingham experience
Source: Postgrad Med J. 2021 Jan 27;97(1150):532–8. doi: 10.1136/postgradmedj-2020-139280 (PMC7843209; doi:10.1136/postgradmedj-2020-139280)
Supplement: postgradmedj-97-532-DC1-inline-supplementary-material-1 [file postgradmedj-97-532-dc1-inline-supplementary-material-1.pdf]

Glossary of terms :

Core surgical trainee: A junior doctor who has usually completed two foundation years of general medical and surgical training after medical school. They spend two core years across different areas of surgery. Historically this role was known as a senior house officer (SHO).

Specialty trainee: A junior doctor who has completed foundation and core training and embarks on a training programme in a particular surgical specialty. This role may also be referred to as a registrar.

Middle grade: A registrar-grade junior doctor who is not on a formal training programme

Peri-CCT fellow: A junior doctor who has completed or is about to complete their specialty training where a certificate of completion of training (CCT) is acquired. They are then eligible to apply for consultant positions.
